# Detailed Anatomy of Bridging Veins Around the Foramen Magnum: a Multicenter Study Using Three-dimensional Angiography

**DOI:** 10.1007/s00062-023-01327-6

**Published:** 2023-08-08

**Authors:** Masafumi Hiramatsu, Tomohiko Ozaki, Shuichi Tanoue, Katsuhiro Mizutani, Hajime Nakamura, Kohei Tokuyama, Hiroyuki Sakata, Yuji Matsumaru, Ichiro Nakahara, Yasunari Niimi, Toshiyuki Fujinaka, Hiro Kiyosue

**Affiliations:** 1 Department of Neurological Surgery, Okayama University Faculty of Medicine, Dentistry and Pharmaceutical Sciences, 2-5-1 Shikata-cho, Kita-ku, Okayama, 700-8558 Japan; 2 Department of Neurosurgery, National Hospital Organization, Osaka National Hospital, Osaka, Japan; 3 Department of Radiology, Kurume University School of Medicine, Kurume, Japan; 4 Department of Neurosurgery, Keio University School of Medicine, Tokyo, Japan; 5 Department of Neurosurgery, Osaka University Graduate School of Medicine, Osaka, Japan; 6 Department of Radiology, Oita University Faculty of Medicine, Yuhu, Japan; 7 Department of Neuroendovascular Therapy, Kohnan Hospital, Sendai, Japan; 8 Division of Stroke Prevention and Treatment, Department of Neurosurgery, Faculty of Medicine, University of Tsukuba, Tsukuba, Japan; 9 Department of Comprehensive Strokology, Fujita Health University School of Medicine, Toyoake, Japan; 10 Department of Neuroendovascular Therapy, St Luke’s International Hospital, Tokyo, Japan; 11 Department of Diagnostic Radiology, Kumamoto University Faculty of Medicine, Kumamoto, Japan

**Keywords:** Bridging vein, Foramen magnum, Cone-beam CT, Venous phase three-dimensional rotational angiography, Slab maximum intensity projection, Dural arteriovenous fistula

## Abstract

**Background and Purpose:**

There has been limited literature regarding the bridging veins (BVs) of the medulla oblongata around the foramen magnum (FM). The present study aims to analyze the normal angioarchitecture of the BVs around the FM using slab MIP images of three-dimensional (3D) angiography.

**Methods:**

We collected 3D angiography data of posterior fossa veins and analyzed the BVs around the FM using slab MIP images. We analyzed the course, outlet, and number of BVs around the FM. We also examined the detection rate and mean diameter of each BV.

**Results:**

Of 57 patients, 55 patients (96%) had any BV. The median number of BVs was two (range: 0–5). The BVs originate from the perimedullary veins and run anterolaterally to join the anterior condylar vein (ACV), inferior petrosal sinus, sigmoid sinus, or jugular bulb, inferolaterally to join the suboccipital cavernous sinus (SCS), laterally or posterolaterally to join the marginal sinus (MS), and posteriorly to join the MS or occipital sinus. We classified BVs into five subtypes according to the draining location: ACV, jugular foramen (JF), MS, SCS, and cerebellomedullary cistern (CMC). ACV, JF, MS, SCS, and CMC BVs were detected in 11 (19%), 18 (32%), 32 (56%), 20 (35%), and 16 (28%) patients, respectively. The mean diameter of the BVs other than CMC was 0.6 mm, and that of CMC BV was 0.8 mm.

**Conclusion:**

Using venous data from 3D angiography, we detected FM BVs in most cases, and the BVs were connected in various directions.

## Introduction

The bridging vein (BV) is a connecting vessel that drains venous blood flow from the brain’s surface to the surrounding dural sinus. There are many BVs around the major dural sinuses, such as the superior sagittal and transverse sinuses. They have been analyzed in detail using various imaging modalities [1, 2]. Venous hypertension due to the dural arteriovenous fistula (dAVF) can cause venous reflux to the cerebral or cerebellar veins via the BVs, leading to venous congestion of the brain parenchyma and intracranial hemorrhage. The BVs of the medulla oblongata have crucial roles as the draining veins of specific dAVFs [3]. There has been limited literature on the BVs of the medulla oblongata around the foramen magnum (FM). Although there have been a few cadaveric studies of BVs around the FM, there has been no comprehensive research on angiographic anatomy. The present study aims to analyze the normal angioarchitecture of the BVs around the FM using slab MIP images of three-dimensional (3D) angiography.

## Methods

All procedures performed in studies involving human participants were performed in accordance with the ethical standards of our institutional research committee. The institutional review boards approved this retrospective study in all collaborative institutions. Written informed consent was obtained from all patients before angiography; however, the need for informed consent for this study was waived because of the retrospective noninvasive study design. Participating centers were selected from among members of the Japanese Society for Neuroendovascular Therapy (JSNET) based on a recognized reputation for treating vascular disorders and an expressed interest in study participation.

### Study Population

We included patients aged 20–80 years admitted to eight participating centers (Affiliation number 1–8) between January 2013 and October 2022 with 3D angiography data of posterior fossa veins. The 3D angiography data of posterior fossa veins included contrast-enhanced cone-beam CT (CE-CBCT) with long acquisition mode and venous phase 3D-rotational angiography (3D-RA) of the dominant side vertebral artery (VA). The CE-CBCT aimed to visualize micro-angioarchitecture such as implanted intracranial stent apposition or vertebral artery dissection. The aim of the venous phase 3D-RA was the preoperative evaluation of vascular disease or intracranial tumor and evaluation of venous disorder. We excluded patients with no venous 3D data of the region of interest, arteriovenous shunt disease, post-craniotomy of the posterior fossa, or insufficient data due to artifact or acquisition timing.

We collected the clinical and radiological data of 85 patients. Clinical characteristics were age, sex, and disease name. Radiological data were angiography in Digital Imaging and Communications in Medicine (DICOM) data format. Among them, 28 patients were excluded from this study based on the exclusion criteria described above: no venous 3D data of region of interest (21 patients), post-craniotomy (3 patients), and insufficient data for analyses (4 patients). The remaining 57 patients (31 males, 26 females; age range, 40–78 years; mean, 58 years) were retrospectively evaluated. All 57 patients had undergone 3D angiography of one-sided vertebral artery; thus, 57 3D data in total were analyzed. The underlying disease was an intracranial aneurysm in 35 patients, intracranial tumor in 12 patients, and other conditions such as suspected venous abnormality or pre-surgical evaluation of microvascular decompression in 10 patients.

### Angiographic Evaluation

**Fig. 1:**
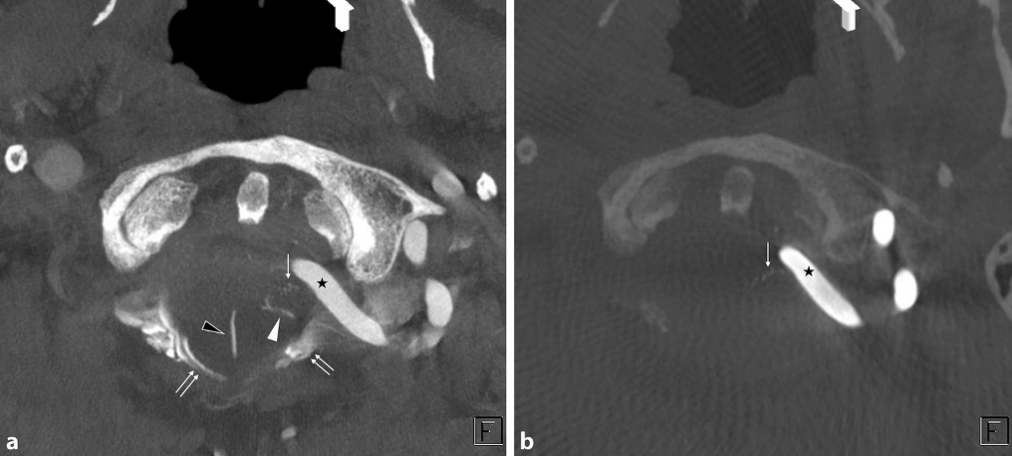
Differentiation between arteries and veins in contrast-enhanced cone-beam CT (CE-CBCT). The axial view of the slab MIP images of CE-CBCT (**a**) and arterial phase three-dimensional rotational angiography (**b**). CE-CBCT with long acquisition mode includes data on arteries and veins. We can only observe arteries (*arrow*) in arterial phase three-dimensional rotational angiography (**b**). Consequently, we can confirm that the other vessels are veins (*white and black arrowheads*) or sinuses (*double arrows*) in CE-CBCT (**a**). *Star* shows the left vertebral artery

Diagnosis was carried out by members of this study group, a panel consisting of nine neurosurgeons and three neuroradiologists, all but one (K.T.) certified as neuro-interventionalists by the JSNET. All collected angiographic data were reviewed using Horos v.3.3.6, a free, open-source medical image viewer. The 3D data of posterior fossa veins were evaluated mainly using slab MIP images from CE-CBCT or venous phase 3D-RA. Because CE-CBCT with long acquisition mode included data on arteries and veins, we distinguished arteries and veins using CE-CBCT and arterial phase 3D-RA data (Fig. 1). We defined the BVs as including the radiculomedullary veins, which run along the cranial and spinal nerve roots. We analyzed the course, outlet, and number of BVs around the FM in each patient. We also examined the detection rate and mean diameter of each BV.

### Statistical Analyses

All calculations were performed using JMP 14 software (SAS Institute, Inc.). Radiological data, including angiographic modality, angiography side, and detection rate of BVs, were summarized using descriptive statistics. We described the number of detectable BVs as the median and interquartile range (IQR). The number of detectable BVs divided into angiography side (right and left) or imaging modality (CE-CBCT and 3D-RA) were assessed using the Wilcoxon signed-rank test. A *p*-value of 0.05 or lower was considered to be statistically significant. The diameter of each BV was described as mean and range.

## Results

The baseline data of angiography were as follows: angiography side was right in 28 patients and left in 29 patients; imaging modality was CE-CBCT in 29 patients and venous phase 3D-RA in 28 patients.

Of 57 patients, 55 patients (96%) had any BV around the FM and a total of 122 BVs were detected. The number of BVs were 0, 1, 2, 3, 4, and 5 in 2, 16, 17, 17, 4, and 1 patients, respectively (median, 2 [IQR, 1–3]). We detected one to five BVs (median, 2 [IQR, 2–3]) in the 3D data of right-side VA angiography and zero to four BVs (median, 2 [IQR, 1–3]) in the 3D data of left-side VA angiography. There was no significant difference in the number of detected BVs according to the laterality of VA angiography (*p* = 0.23). We detected zero to five BVs (median, 2 [IQR, 2–3]) in CE-CBCT data and one to four BVs (median, 2 [IQR, 1–3]) in venous phase 3D-RA. There was no significant difference in the number of detected BVs according to the imaging modality (*p* = 0.13).

### Course and Terminations of BVs

**Fig. 2:**
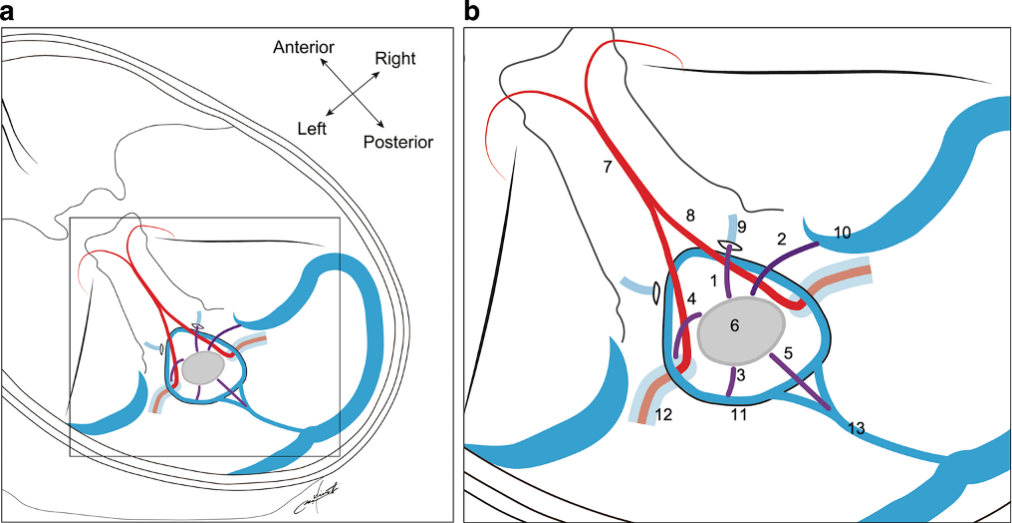
Schematic illustration (**a**) and its partly expanded illustration (**b**) of bridging veins (BVs) and surrounding vessels around the foramen magnum: *1* indicates the anterior condylar vein (ACV) BV; *2*, jugular foramen (JF) BV; *3*, marginal sinus (MS) BV; *4*, suboccipital cavernous sinus (SCS) BV; *5*, cerebellomedullary cistern (CMC) BV; *6*, cross-section of the medulla oblongata; *7*, basilar artery; *8*, vertebral artery (VA); *9*, ACV in the hypoglossal canal; *10*, jugular bulb or sigmoid sinus; *11*, MS; *12*, SCS around the extracranial VA; *13*, occipital sinus

**Fig. 3:**
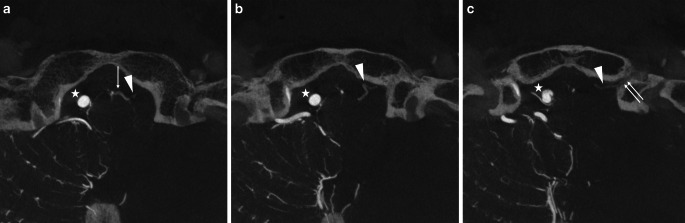
A representative case of the anterior condylar vein (ACV) bridging vein (BV). Axial view of the slab MIP images from contrast-enhanced cone-beam CT shows that the transverse medullary vein (*arrow* in (**a**)) drains into the ACV (*double arrows* in (**c**)) in the hypoglossal canal via the ACV BV (*arrowheads* in (**a**)–(**c**)). *Star* shows the right vertebral artery

**Fig. 4:**
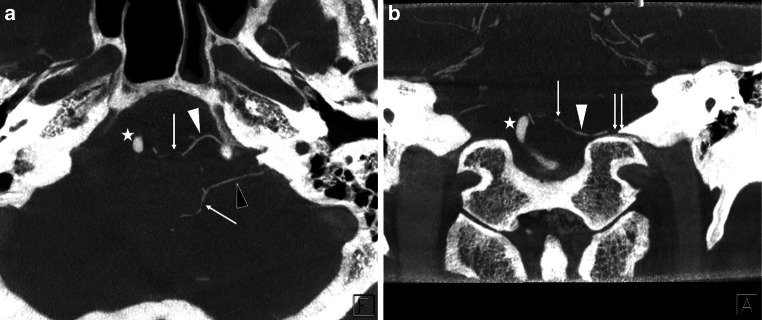
A representative case of the jugular foramen (JF) bridging vein (BV). Axial (**a**) and coronal (**b**) view of the slab MIP images from contrast-enhanced cone-beam CT show that the transverse medullary vein (*arrow* in (**a**) and (**b**)) drains into the inferior petrosal sinus (*double* *arrows* in (**b**)) and sigmoid sinus (not shown) via two JF BVs (*white and black arrowheads*). *Star* shows the left vertebral artery running close to the opposite side

**Fig. 5:**
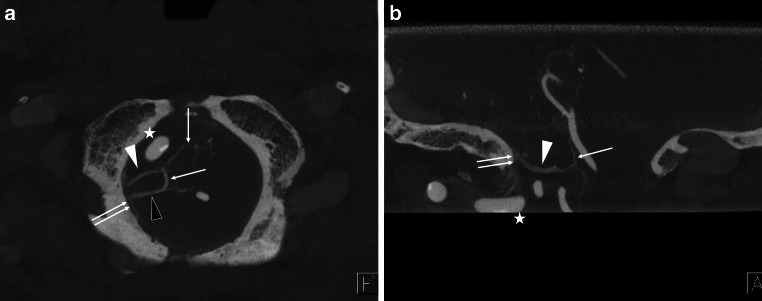
A representative case of the marginal sinus (MS) bridging vein (BV). Axial (**a**) and coronal (**b**) view of the slab MIP images from contrast-enhanced cone-beam CT show that the transverse medullary vein (*arrow* in (**a**)) and lateral medullary vein (*arrow* in (**b**)) drains into the MS (*double* *arrows* in (**a**) and (**b**)) via two MS BVs (*white and black arrowheads*). *Star* shows the right vertebral artery

**Fig. 6:**
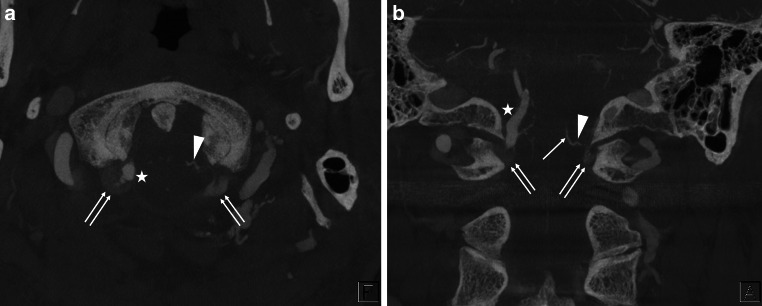
A representative case of the suboccipital cavernous sinus (SCS) bridging vein (BV). Axial (**a**) and coronal (**b**) view of the slab MIP images from contrast-enhanced cone-beam CT show that the lateral medullary vein (*arrow* in (**b**)) drains into the SCS (*double* *arrows* in (**a**) and (**b**)) via a SCS BV (*arrowhead*). *Star* shows the right vertebral artery

**Fig. 7:**
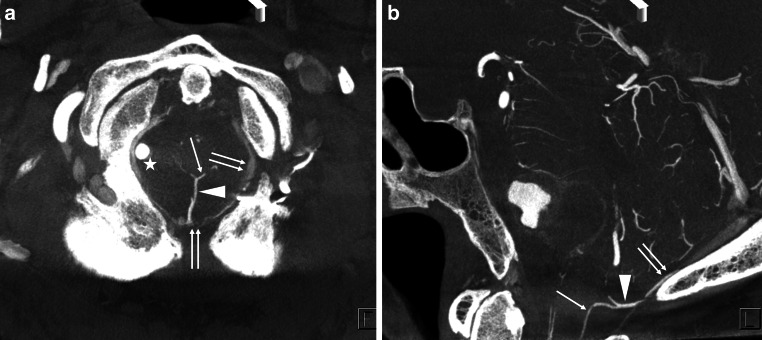
A representative case of the cerebellomedullary cistern (CMC) bridging vein (BV). Axial (**a**) and sagittal (**b**) view of the slab MIP images from contrast-enhanced cone-beam CT show that the transverse medullary vein (*arrow* in (**a**)) and posterior spinal vein (*arrow* in (**b**)) drain into the marginal sinus (*double* *arrows* in (**a**) and (**b**)) via a CMC BV (*arrowhead*), which is the posteromedian BV running through the CMC. *Star* shows the right vertebral artery

The BVs originate from the perimedullary veins and run anterolaterally to join the anterior condylar vein (ACV), inferior petrosal sinus, sigmoid sinus, or jugular bulb, inferolaterally to join the suboccipital cavernous sinus (SCS), laterally or posterolaterally to join the marginal sinus (MS), and posteriorly to join the MS or occipital sinus. We classified BVs around the FM into five subtypes based on the draining pattern into the surrounding venous sinus or vein (Fig. 2):

1. The ACV BV empties into the ACV (Fig. 3).
2. The jugular foramen (JF) BV empties into the inferior petrosal sinus, sigmoid sinus, or jugular bulb (Fig. 4).
3. The MS BV empties into the MS other than the posteromedian direction (Fig. 5).
4. The SCS BV empties into the SCS (Fig. 6).
5. The cerebellomedullary cistern (CMC) BV is a posteromedian BV emptying into the MS or occipital sinus (Fig. 7).

Based on our classification of BVs around the FM, among the total of 122 BVs, 12 BVs were ACV BV, 24 were JF BV, 45 were MS BV, 25 were SCS BV, and 16 were CMC BV.

### Detection Rate and Diameter of Each BV

Among the 57 patients, we calculated the detection rate of each BV divided into the ipsilateral and contralateral side to VA angiography except for the CMC BV located midline (Table 1). On the ipsilateral side of VA angiography, ACV, JF, MS, and SCS BVs were detected in 4 (7%), 15 (26%), 23 (40%), and 13 patients (23%), respectively. On the contralateral side of VA angiography, ACV, JF, MS, and SCS BVs were detected in 8 (14%), 7 (12%), 16 (28%), and 10 patients (18%), respectively. On any side, ACV, JF, MS, and SCS BVs were detected in 11 (19%), 18 (32%), 32 (56%), and 20 patients (35%), respectively. The CMC BV was detected in 16 patients (28%). Any BV was detected on any side in 55 patients (96%). There were 10 cases (MS, 6; JF, 2; SCS, 2) with two same-subtype BVs on the same side. The mean diameters (range) of ACV, JF, MS, SCS, and CMC BVs were 0.6 (0.4–0.9) mm, 0.6 (0.3–0.9) mm, 0.6 (0.3–1.2) mm, 0.6 (0.3–0.9) mm, and 0.8 (0.5–1.3) mm, respectively.

**Table 1:** Detection rate of each bridging vein around the foramen magnum

	Ipsilateral to VAG (%)	Contralateral to VAG (%)	Any side (%)
ACV BV	4 (7 )	8 (14)	11 (19)
JF BV	15 (26)	7 (12)	18 (32)
MS BV	23 (40)	16 (28)	32 (56)
SCS BV	13 (23)	10 (18)	20 (35)
CMC BV	–	–	16 (28)
All BVs	–	–	55 (96)

*ACV* anterior condylar vein; *BV* bridging vein; *CMC* cerebellomedullary cistern; *JF* jugular foramen; *MS* marginal sinus; *SCS* suboccipital cavernous sinus; *VAG* vertebral angiography

## Discussion

In the present study, we demonstrated that a total of 122 BVs were detected (median, 2 per patient) and that 55 patients (96%) had any BVs around the FM. We classified the BVs into five subtypes based on the draining pattern. The MS BV was the most frequent, and the CMC BV was the largest.

Historically, supratentorial and cerebellar BVs have been systematically investigated in cadaveric and radiological studies [1, 2]. Rhoton divided the BVs of the posterior fossa into four groups: the galenic group draining into the vein of Galen; the tentorial group draining into the torcular and tentorial sinus; the petrosal group draining into the petrosal sinus; and other BVs [4]. In this article, some BVs around the FM have been listed as one of the “other” infrequent BVs. Baltsavias et al. summarized medullary BVs in their review article [5]. Although they described BVs draining into the inferior petrosal sinus, sigmoid sinus, jugular bulb, MS, ACV, and occipital sinus, the description was not carried out in a systematic manner.

Duvernoy reported detailed venous anatomy around the brainstem based on 50 cadaver brains [6]. He mainly described the BVs as the “satellite veins of the cranial nerves,” not as BVs. He demonstrated the satellite veins running alongside the ninth to twelfth cranial nerves and the first cervical nerve root in figures. The satellite veins of the glossopharyngeal and vagal nerve drained into the inferior petrosal sinus around the JF and were called the inferior petrosal vein. These veins may represent the JF BV in the present study. Similarly, the satellite vein of the hypoglossal nerve is thought to be the ACV BV, and the satellite vein of the first cervical nerve root could be the SCS BV. Duvernoy reported that the frequency of the satellite veins described as the glossopharyngeal nerve, hypoglossal nerve, and first cervical nerve root was 30, 8, and 32%, respectively.

Duvernoy also reported the vein of the cerebellomedullary cistern. The posteromedian medullary vein drains downward into the MS through the vein of the cerebellomedullary cistern, which is the same vein as our CMC BV. He reported that the frequency of this vein was 50%. Matsushima also reported detailed venous anatomy of the BVs based on 25 cadaver brains [7]. The BVs drained into the inferior petrosal sinus near the JF, and the inferior part of the sigmoid sinus appeared in 8.3 and 37.5% cadavers. We assume that the JF BV in the present study represents these veins by Matsushima. Matsushima also demonstrated that another BV that drained into the MS around the FM appeared in 41.7% of cadavers, which may represent the MS BV. Kiyosue et al. reported the anterior medullary-anterior pontomesencephalic venous system and its BVs based on gadolinium-enhanced MRI in 35 patients [8]. They identified BVs connecting to the SCS in 11 cases (31%), inferior petrosal sinus in 5 cases (14%), MS in 3 cases (9%), and jugular bulb in 1 case.

In the spinal region, there are two kinds of drainage veins from the spinal cord: radiculomedullary veins and BVs. The radiculomedullary veins run along the nerve root and pierce the dura at the spinal nerve root sleeve. The BVs of the spinal cord run apart from the nerve root and pierce the dura independently of the nerve root sleeve. We believe that the satellite veins described by Duvernoy [7] correspond to the radiculomedullary veins; hence, some ACV, JF, and SCS BVs are radiculomedullary veins. However, the MS and CMC BVs correspond to the BVs of the spinal cord because they run apart from the cranial nerve root. It was challenging to differentiate between radiculomedullary veins and BVs because there are no data regarding the nerve root in the collected data in the present study. Therefore, we defined the BVs as including the radiculomedullary veins in this study.

We summarized the frequency of each BV in the published literature and our results in Table 2. In the present study, the frequency of each BV is higher than in the MRI study. This difference is due to the use of images with higher spatial resolution, which increases the detection rate and makes it comparable to cadaver studies. As shown in Table 2, the published literature has only partially demonstrated the frequency of BVs, but we can systematically assess the frequency in the present study. Moreover, the current research has the largest number of study subjects.

**Table 2:** Summary of reported detection rate of bridging veins around the foramen magnum

Author	Method	Number	ACV BV (in %)	JF BV (in %)	MS BV (in %)	SCS BV (in %)	CMC BV (in %)
Duvernoy	Cadaver	50	8	> 30	*n*. d.	32	50
Matsushima	Cadaver	25	*n*. d.	45.8	41.7	*n*. d.	*n*. d.
Kiyosue	Gd-MRI	35	*n*. d.	17	9	31	*n*. d.
Present study	3D-RA CBCT	57	19	32	56	35	28

*ACV* anterior condylar vein; *BV* bridging vein; *CBCT* cone-beam CT; *CMC* cerebellomedullary cistern; *Gd* Gadolinium; *JF* jugular foramen; *MS* marginal sinus; *SCS* suboccipital cavernous sinus; *3D-RA* 3D rotational angiography

### Clinical Implications

BVs around the FM may be involved in dAVF as draining veins. Mitsuhashi et al. reported dAVFs draining into the petrosal vein or BV of the medulla [3]. They described six patients with dAVF draining into the BVs of the medulla, and all of them were dAVFs of the FM or craniocervical junction (CCJ). They also described these dAVFs as having male predominance and a higher incidence of aggressive neurological manifestations than dAVFs of the transverse-sigmoid or cavernous sinus. Recently, angioarchitecture and characteristics of CCJ and FM dAVF have been analyzed in detail [9, 10]. Most CCJ and FM dAVF involve the BVs around the FM. Based on the anatomical location, the CCJ dAVF in the occipital-C1 level may involve the SCS BV, and the FM dAVF may involve the other types of BVs. Spittau et al. reviewed the dAVFs of the hypoglossal canal. They classified them into three types in terms of their dominant venous drainage: Type 1, anterograde; Type 2, retrograde orbital; Type 3, pial or perimedullary [11]. The dAVF of the hypoglossal canal with perimedullary drainage may involve the ACV BV. Kawasaki et al. showed a hypoglossal canal dAVF with venous reflux to the medulla oblongata via the ACV BV using slab MIP images [12]. Byun et al. reported a case of JF dAVF with venous reflux to the brainstem via the BV, which was assumed to be JF BV [13]. Sugiura et al. reported sigmoid sinus dAVF with venous reflux to the medulla oblongata via the JF BV [14]. Tanaka et al. reported the occipital sinus dAVF with venous reflux to the medulla oblongata via the CMC BV [15]. Knowledge of these BVs helps us understand angioarchitecture in these complicated dAVF around the FM.

### Study Limitations

There were some study limitations. First, we could not clearly identify the dural sinus to which the BVs connect in some cases. Second, due to the perfusion area of the VA, the contralateral side is not sufficiently opacified with contrast media for evaluation in some cases. Although CT angiography or enhanced MRI can solve these problems, the present study has the advantage of angiographic images of the dominant side VA with higher spatial resolution, which increases the detection rate.

### Conclusions

FM BVs are frequently observed. Because of the rich surrounding venous network, they drain in various terminations.

## Abbreviations

3D-RA: Three-dimensional rotational angiography
ACV: Anterior condylar vein
BV: Bridging vein
CCJ: Craniocervical junction
CE-CBCT: Contrast-enhanced cone-beam CT
CMC: Cerebellomedullary cistern
DAVF: Dural arteriovenous fistula
FM: Foramen magnum
IQR: Interquartile range
JF: Jugular foramen
MS: Marginal sinus
SCS: Suboccipital cavernous sinus
VA: Vertebral artery
